# Editorial: Sleep in children with rare disorders: Having a sleep problem is not that rare

**DOI:** 10.3389/fneur.2022.1070414

**Published:** 2022-11-14

**Authors:** Karen Spruyt

**Affiliations:** Université de Paris, NeuroDiderot-INSERM, Paris, France

**Keywords:** rare disorder, sleep, circadian, questionnaire, melatonin, sleep monitoring devices

Any disease that affects a small percentage of the population is considered rare. Yet, no exact cut-off exists to determine rareness. The six papers included in this Research Topic, however, demonstrate that sleep problems are not rare in those diseases that affect only a few of us.

Although it was not the scope of this Research Topic to focus on one rare disorder, three articles brought sleep issues of individuals with Rett syndrome into the spotlight. Rett syndrome is a severe X-linked developmental brain disorder almost exclusively affecting females. For more than 95% of classical Rett cases, *de novo* mutations in the Methyl-CpG Binding protein 2 (*MECP2*) gene on the long arm of X chromosome is responsible. Whereas mutations in the cyclin-dependent kinase-like 5 (*CDKL5*) and the forkhead box G1 (*FOXG1*) are responsible for atypical Rett cases. Tascini et al. describe in length the pathophysiology and clinical features framing the complexity of the rare disease, and as such its multiple disabilities. Poor sleep, including difficulties breathing and seizures, are at the core of most sleep findings being summarized. Leoncini et al. zoom in on such breathing abnormalities. It is indeed an ongoing discussion whether or not Rett cases have respiratory abnormalities during their sleep, and how this relates to their characteristic breathing dysfunction during wakefulness. In 66 cases (age range 2–32 years old) with a clinical diagnosis of typical RTT and proven *MECP2* gene mutation, a portable polygraphic recording of cardiorespiratory parameters was performed. An Apnea-hypopnea index (AHI) > 15 was considered clinically significant, whilst the objective was to examine the oxidative stress markers. Although evidence of oxidative stress was found, and appeals replication, their finding on apneas underlines the complexity of breathing abnormalities in rare disorders such as Rett syndrome. Namely, those with sleep apneas demonstrated AHI > 15, higher total breath-holding episodes, and lower nadir SpO_2_, whilst those with apneas during wakefulness showed a lower average SpO_2_. Taken together, both articles on Rett cases pinpoint the need for sleep studies. Such sleep studies may foster pathophysiologic hypotheses that can be mimicked in animal studies and ultimately lead to therapeutical interventions. Providing an overview, and as such a benchmark for future studies, was the scope of Zhang and Spruyt, in their meta-review of standard sleep study parameters collected through sleep studies of Rett cases. Overall, 13 studies reporting on 134 Rett cases were summarized, as a group and along strata by gene, age, and clinical features. Increased stage NREM 3 and decreased REM sleep were found, and thus results are in concordance with sleep literature reporting on cases with intellectual disabilities. Sleep-disordered breathing was as well confirmed yet given that findings are generated from clinical papers (hence clinical referral), a publication bias should be considered. This meta-review further highlights that perturbed sleep in Rett cases may vary with the presence of epilepsy and age.

Age is an important variable in research, particularly in sleep research. That is, for instance, during puberty circadian preferences are manifesting. Martinez-Cayuelas et al. explored the combination of an ambulatory circadian monitoring (ACM) device and saliva samples to determine dim light melatonin onset (DLMO) in autistic children (*n* = 41, 9.9 ± 3.02 years old). Their study tackles two bottlenecks in sleep research for those with developmental disabilities, namely, the struggle to measure sleep and circadian preferences with minimal discomfort, and to treat those with sleep difficulties (e.g., exogenous melatonin). Such a struggle was also made clear through the objective of Fetta et al.. In Pallister-Killian syndrome, a rare genetic disorder with multi-organ involvement caused by mosaic tetrasomy of chromosome 12p (*n* = 14, 1–17 years old), a combination of 24-h video-polysomnography and the Sleep Disturbance Scale for Children were applied. The findings of these two studies were that DLMO timing occurred later in autistic children and that disorders of initiating and maintaining sleep as well as sleep-disordered breathing were omnipresent in cases with Pallister-Killian syndrome. Additionally, both studies suggest that the applied tools are applicable, but foremost their studies are an innovative contribution to the literature through their explorative nature in terms of tools applied and sleep-circadian objective. Thus far, five of the six studies included in this Research Topic mark the importance of sleep studies, sleep architecture, and sleep-related features (such as breathing, epilepsy, and melatonin secretion). The review of 32 articles on childhood craniopharyngioma, the most common non-neuroepithelial intracerebral neoplasm in children, teaches us about hypothalamic involvement. The hypothalamus is in control of our bodily homeostasis, particularly the sleep-wake regulatory system. Cordani et al. displayed the wide spread of sleep problems reported: narcolepsy was reported in 14–35% and sleep-disordered breathing in 4–46%. But likely the most striking finding, and one that should not be overlooked in samples with complex clinical profiles, is somnolence. Depending on the diagnostic method of detection, prevalence rates of 25–43% by subjective measures, and 50–100% by objective investigations were found. In short, these six articles comprise all aspects of sleep health and at the same time indicate the dire emergency to address poor sleep of those individuals with a rare disorder.

Altogether, each of these articles call attention to sleep issues, which are not so rare. The articles further articulate the bottlenecks in measuring (1), diagnosing, and treating sleep problems in individuals affected by a rare disorder. Indirectly each of the studies included, and also the reviewed studies, emphasize the utmost need for management kits. A non-pharmacological approach is potentially the most powerful first-line intervention for the cases as well as the caregiver/caretaker. Whereas investigations of the sleep macrostructure and coexisting conditions such as epilepsy, and breathing abnormalities during the sleep state in individuals with a rare disorder may foster pharmaceutical or machine-driven approaches.

We hope that this Research Topic, with a mixture of original research and review articles, demonstrated that rare diseases are rare but individuals with rare disorders are not considering their sleep problems. In individuals with rare disorders, their sleep issues should no longer be treated as a comorbid or co-occurring problem. Furthermore, their uniqueness may shed a new light on sleep-wake regulation in the general population.

Rare cases are special, not in terms of their needs but by their contribution to insights into sleep-wake regulation, and its perturbations. Let the work presented in this Research Topic be an invitation for more research on sleep in children with rare disorders, and foremost an expression of gratitude to those willing to participate in our sleep research.

## Author contributions

The author confirms being the sole contributor of this work and has approved it for publication.

## Conflict of interest

The author declares that the research was conducted in the absence of any commercial or financial relationships that could be construed as a potential conflict of interest.

## Publisher's note

All claims expressed in this article are solely those of the authors and do not necessarily represent those of their affiliated organizations, or those of the publisher, the editors and the reviewers. Any product that may be evaluated in this article, or claim that may be made by its manufacturer, is not guaranteed or endorsed by the publisher.
